# Peri-Ictal Autonomic Control of Cardiac Function and Seizure-Induced Death

**DOI:** 10.3389/fnins.2021.795145

**Published:** 2022-01-21

**Authors:** Ian C. Wenker, Elizabeth A. Blizzard, Pravin K. Wagley, Manoj K. Patel

**Affiliations:** Department of Anesthesiology, University of Virginia, Charlottesville, VA, United States

**Keywords:** SUDEP (sudden unexplained death in epilepsy), epilepsy, bradycardia, seizure, apnea, *SCN8A* encephalopathy, tonic phase

## Abstract

Sudden unexpected death in epilepsy (SUDEP) accounts for the deaths of 8–17% of patients with epilepsy. Although the mechanisms of SUDEP are unknown, one proposed mechanism is abnormal control of the heart by the autonomic nervous system (ANS). Our objective was to determine whether the broad changes in ictal heart rate experienced by mouse models of SUDEP are (1) due to the ANS and (2) contribute to seizure-induced death. Seizures were induced by electrical stimulation of the hippocampus of a mouse carrying the human *SCN8A* encephalopathy mutation p.Asn1768Asp (N1768D; “D/+ mice”). Using standard autonomic pharmacology, the relative roles of the parasympathetic and sympathetic nervous systems on heart rate changes associated with seizures were determined. All induced seizures had pronounced ictal bradycardia and postictal tachycardia. Seizure susceptibility or severity were unchanged by the pharmacological agents. Administration of Atropine, a muscarinic antagonist, eliminated ictal bradycardia, while carbachol, a muscarinic agonist, had no effect on ictal bradycardia, but reduced postictal tachycardia. Sotalol, an adrenergic β-receptor antagonist, had no effect on ictal bradycardia, but did suppress postictal tachycardia. Isoproterenol, a β-receptor agonist, had no effect on either ictal bradycardia or postictal tachycardia. Administration of the α1-receptor antagonist prazosin increases the incidence of seizure-induced death in D/+ mice. Although postictal heart rate was lower for these fatal seizures in the presence of prazosin, rates were not as low as that recorded for carbachol treated mice, which all survived. Both ictal bradycardia and postictal tachycardia are manifestations of the ANS. Bradycardia is mediated by a maximal activation of the parasympathetic arm of the ANS, and tachycardia is mediated by parasympathetic inactivation and sympathetic activation. While the changes in heart rate during seizures are profound, suppression of postictal heart rate did not increase seizure mortality.

## Introduction

Sudden unexpected death in epilepsy (SUDEP) is defined as the sudden, unexpected, non-traumatic, and non-drowning death of a person with epilepsy for which postmortem examination does not reveal another cause of death (Nashef et al., 2012). SUDEP is the most common cause of death associated with epilepsy, accounting for between 8 and 17% of all epilepsy-related deaths (Terra et al., 2013). This number can increase to 50% in patients with poorly controlled seizures (Devinsky, 2011; Tolstykh and Cavazos, 2013).

Considering the diverse patient population affected, SUDEP is likely influenced by multiple factors; however, in all cases the final event that causes death is likely to be cardiac and/or respiratory failure. Cardiac dysfunction has long been considered a likely cause of death, as arrhythmias are the cause of many other forms of sudden death (Huikuri et al., 2001; Srinivasan and Schilling, 2018), cardiac rhythm disturbances are often associated with seizures (Schraeder and Lathers, 1989; Nashef et al., 1996; Langan et al., 2000; So et al., 2000), and some long QT gene mutations have been linked to SUDEP (Johnson et al., 2009; Tiron et al., 2015; Auerbach et al., 2016; Smith, 2016).

In the study known as MORTEMUS, in which several patients who died from convulsive seizures had cardiorespiratory recordings, it was observed that bradycardia preceded death (Ryvlin et al., 2013). Bradycardia during and after fatal seizures has also been observed in animal models of epilepsy, including in anesthetized cats (Wasterlain, 1974), and a number of mouse models of epilepsy (Kalume et al., 2013; Aiba and Noebels, 2015; Aiba et al., 2016; Nakase et al., 2016; Kim et al., 2018; Loonen et al., 2019), including mice with *Scn8a* mutations used in the present study (Frasier et al., 2016; Wengert et al., 2021; Wenker et al., 2021).

The central nervous system exercises significant control of cardiac function *via* both branches of the ANS: the sympathetic and parasympathetic. Ictal bradycardia has been observed in a number of animal models of epilepsy (Wasterlain, 1974; Doba et al., 1975; Goodman et al., 1990, 1999; Kalume et al., 2013; Aiba and Noebels, 2015; Aiba et al., 2016; Kim et al., 2018; Dhaibar et al., 2019; Loonen et al., 2019; Wengert et al., 2021; Wenker et al., 2021). The use of muscarinic antagonists has demonstrated that this bradycardia is due to activation of the parasympathetic nervous system in anesthetized cats (Doba et al., 1975) and in kindled rats (Goodman et al., 1990, 1999).

However, very few studies have directly addressed the role of the ANS during seizures in the context of SUDEP. In mouse models of Dravet Syndrome—a form of pediatric epilepsy with high incidence of SUDEP (Dravet, 2011; Sakauchi et al., 2011)—atropine prevented bradycardia during thermally induced fatal seizures (Kalume et al., 2013; Kim et al., 2018). However, atropine also prevented death in these mice, even when administered intracerebroventricularly, making it is difficult to know whether atropine’s prevention of bradycardia was due to inhibition of parasympathetic input to the heart or some other mechanism.

In the present study, we use a mouse model of SUDEP in which mice harbor a mutation in the gene *Scn8a* (Wagnon et al., 2015). We have previously demonstrated that these mice experience tonic seizures with ictal bradycardia, which if continues postictal, results in fatality (Wengert et al., 2021; Wenker et al., 2021). We utilize the ability to stimulate non-fatal seizures on command that are phenotypically nearly identical to the often-fatal spontaneous seizures. We hypothesized that ictal bradycardia was due to stimulation of the parasympathetic nervous system and postictal tachycardia was due to simultaneous reduction of the parasympathetic and stimulation of the sympathetic arms of the ANS. Using standard pharmacology of the ANS, we demonstrate that (1) ictal bradycardia is due to maximal activation of the parasympathetic innervation of the heart; (2) postictal tachycardia is due to simultaneous parasympathetic reduction and sympathetic stimulation; and (3) while continued bradycardia occurs after a fatal seizure, pharmacologically induced bradycardia is not sufficient to cause seizures to become fatal.

## Materials and Methods

### Mouse Husbandry and Genotyping

All mice were cared for and used in accordance with the Animal Care and Use Committee standards of the University of Virginia (Protocol #3308). Mice were housed in a temperature and humidity-controlled vivarium with a standard 12-h light/dark cycle with food and water *ad libitum*. A colony of transgenic mice harboring the N1768D mutation in a single *Scn8a* allele were maintained by breeding mice heterozygous for the mutation (D/+) with C57BL/6J mice (Jax #000664). D/+ mice have increased mortality, with about half experiencing SUDEP prior to 10 months of age, and are reported to have 0–3 seizures per day (Wagnon et al., 2015). A total of 15 resultant D/+ mice were used for all experiments. Both male and female D/+ mice were used in roughly equal numbers between the ages of 8–10 weeks, and no sex differences were observed for any of the experiments. Genotyping of D/+ mice was performed as previously described (Wagnon et al., 2015).

### Surgical Preparation

Custom electrocorticogram (ECoG)/electrocardiogram (ECG) headsets (PlasticsOne, Inc., or Pinnacle Technology Inc.) were implanted in 6–10-week-old D/+ mice using standard aseptic surgical techniques as we have done previously (Wengert et al., 2021; Wenker et al., 2021). Anesthesia was induced with 5% and maintained with 0.5–3% isoflurane. Adequacy of anesthesia was assessed by lack of toe-pinch reflex. A midline skin incision was made over the skull, and burr holes were made at the lateral/rostral end of both the left and right parietal bones to place EEG leads, and at the interparietal bone for reference electrode. Two twisted wire leads were implanted into the left hippocampus at coordinates 3 mm caudal, 3 mm lateral, and 3 mm ventral of bregma. Two ECG leads were passed subcutaneously to the left abdomen and right shoulder and sutured into place to approximate a lead II arrangement. The headsets were attached to the skull with dental acrylic (Jet Acrylic; Lang Dental). Mice received postoperative analgesia with ketoprofen (5 mg/kg, i.p.) and 0.9% saline (0.5 ml i.p.) and were allowed to recover a minimum of 5 days prior to experiments.

### Recording of ECoG, ECG, and Breathing

Recording of ECoG, ECG, and breathing was performed as previously described (Wengert et al., 2021; Wenker et al., 2021). Plethysmography chambers were built to comply with requirements for continuous housing described in the Guide for the Care and Use of Laboratory Animals (Council, 2011). The floor of the chambers had approximate dimensions of 4.5 × 4.5 inches (>20 sq. inches) and 7 inches tall. There were ports for air in and air out, and for pressure monitoring. The chamber was supplied with a continuous flow of room air at approximately 400 ml/min *via* supply and exhaust air pumps (MK-1504 Aquarium Air Pump; AQUA Culture) balanced to maintain chamber pressure near atmospheric. Mice had access to a continuous supply of water and food. The surgically implanted headsets were attached to a custom low torque swivel cable, allowing mice to move freely in the chamber. To assess breathing frequency, the pressure of the EMU chamber was measured with an analog pressure transducer (SDP1000-L05; Sensirion). ECoG and ECG signals were amplified at 2000 and bandpass filtered between 0.3–100 and 30–300 Hz, respectively, with an analog amplifier (Neurodata Model 12, Grass Instruments Co.). Biosignals were digitized with a Powerlab 16/35 and recorded using LabChart 7 software (AD Instruments, Inc.) at 1 kS/s. Video acquisition was performed by multiplexing four miniature night vision-enabled cameras and then digitizing the video feed with a Dazzle Video Capture Device (Corel, Inc.) and recording at 30 fps with LabChart 7 software in tandem with biosignals.

### Spontaneous and Stimulated Seizure Recording

**FIGURE 1:**
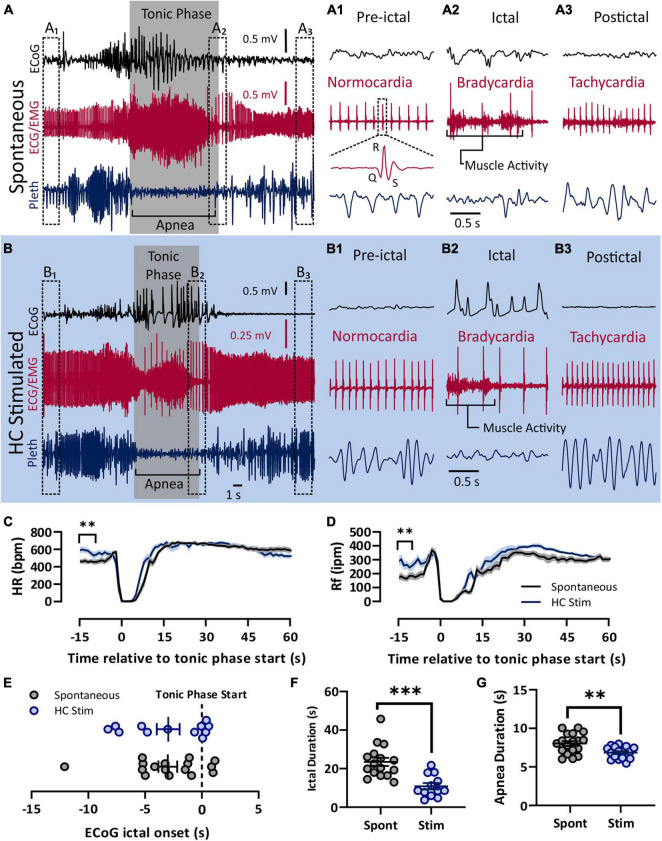
Spontaneous and electrically induced seizures have similar semiology. Both spontaneous **(A)** and HC stimulated **(B)** seizures involved cortical spike-wave discharges, a prolonged tonic phase noted by large amounts of EMG activity, apnea, and bradycardia. **(A)** Recording of ECoG, ECG, and plethyismography (pleth) from a D/+ mouse during a spontaneously occurring seizure. **(A1–A3)** Prior to the spontaneous seizure, heart rate was normal (∼500 bpm; **A1**). Toward the end of the seizure, R-waves can be identified above the muscle activity and bradycardia is apparent **(A2)**. After the seizure, tachycardia relative to the pre-ictal period is observed **(A3)**. Y-axis scale for **(A1–A3)** is the same as for **(A)** and time scale depicted in **(A2)** is the same for **(A1,A3)**. **(B)** Identical semiology is observed during seizures due to electrical stimulation of the hippocampus (HC). **(B1–B3)** HC stimulated seizures also experienced bradycardia during and tachycardia after the seizure. *Y*-axis scale for **(B1–B3)** is the same as for **(B)** and time scale depicted in **(B2)** is the same for **(B1,B3)**. At total of 17 spontaneous seizure were recorded from 4 mice (data in black) and 18 electrically stimulated seizures were recorded from 9 mice (data in blue). **(C)** Heart rate decreased sharply during both spontaneous and HC stimulated seizures. Pre-ictal heart rate was higher for HC stimulated seizures (*p* = 0.0010, *t* = 3.593, df = 33, *d* = 1.22). **(D)** Similarly, breathing ceased during both types of seizures, but pre-ictal breathing for HC stimulates seizures was elevated compared to the same time period of spontaneous seizures (*p* = 0.0048, *u* = 69, *d* = 0.98). **(E)** No difference in timing of the tonic phase relative to ECoG ictal activity (*p* = 0.9818, *u* = 95). **(F)** Compared to spontaneous seizures, HC stimulated seizures (Stim) were shorter in duration (*p* = 0.0001, *u* = 18, *d* = 1.74), and **(G)** had longer apnea duration (*p* = 0.0029, unpaired *t*-test, *t* = 3.222, df = 33, *d* = 1.10). **p < 0.01 and ***p < 0.001.

Four 8–10 week-old D/+ mice were used for chronic recording of seizures. The mice were housed in the plethysmography chambers 24 h a day and provided with water and food *ad libitum* as we have previous described (Wengert et al., 2021; Wenker et al., 2021). A separate cohort of eleven 8–10 week-old D/+ mice were used to induce seizures on command *via* stimulation of two hippocampal leads connected to an isolated pulse stimulator (Model 2100, A-M Systems, Inc.). Mice to be simulated were placed in the chamber 30–60 min prior to stimulation. Stimulations were 2 s trains of 1 ms biphasic current pulses at 50 Hz. Stimulations were repeated every 60 s with increased current amplitude (10–600 μA) until a seizure was produced. The final current amplitude that produced a seizure was recorded as the after-discharge threshold (ADT) for that stimulation. All stimulated seizures were performed between 10 a.m. and 3 p.m. and mice were allowed at least 2 recovery days in between seizure stimulations. All spontaneous and stimulated seizures were behaviorally and electrographically tonic seizures, as previously described for spontaneous seizures in these mice (Wengert et al., 2021; Wenker et al., 2021), and as depicted in Figure 1.

### Breathing and Heart Rate Detection

Individual breaths and heart beats were identified as inspiratory deflections in the pressure transducer signal and R waves in the ECG signal, respectively, using Spike2 software (Cambridge Electronic Design, Ltd.), as previously reported (Wengert et al., 2021; Wenker et al., 2021). Briefly, a breath was scored when the downward deflection went below a certain hysteresis value determined by the experimenter and rose back above a threshold of 0 mV. The minimum time between breaths was set to 0.05 s. Similarly, an R wave was identified when an upward deflection crossed a threshold value determined by the experimenter. The minimum time between R waves was set to 0.02 s. All breaths and R waves were verified by the experimenter.

### Heart Rate Variability Analysis

Sequential RR intervals were exported from Spike2 and imported into MATLAB for analysis as previously described (Piskorski and Guzik, 2007). Poincare plots (examples in Supplementary Figure 1) were made for every minute of recording and geometric mean standard deviations (SD1 and SD2) values.

Equations used for SD1 and SD2 were:

$$S\,D\,1=\sqrt{\frac{1}{n}\,\sum_{i=1}^{n}{\left({\left(\left|\left(x_{i}-x_{C}\right)-\left(y_{i}-y_{C}\right)\right|/\sqrt{2}\right)}_{i}^{1}\right)}^{2}}\,\text{and}$$

$$S\,D\,2=\sqrt{\frac{1}{n}\,\sum_{i=1}^{n}{\left({\left(\left|\left(x_{i}-x_{C}\right)+\left(y_{i}-y_{C}\right)\right|/\sqrt{2}\right)}_{i}^{1}\right)}^{2}},$$

where *n* = total number of RR intervals, x_*c*_ = mean RR_*n*_, and y_*c*_ = mean RR_*n*+1_.

### Pharmacology

All chemicals were purchased from Sigma Aldrich and were either of pharmaceutical grade or were sterile filtered prior to injection. Injections were given intraperitoneal in a volume of 5 μl sterile saline per mg of mouse weight (e.g., 0.1 ml for a 20 mg adult mouse). The concentration of each drug was based on the achieving the desired dosages of 1 mg/kg atropine, 0.5 mg/kg carbachol Cl, and 10 mg/kg sotalol HCl, 50 mg/kg isoproterenol HCl, and 1 mg/kg prazosin HCl. Injection of 5 μl sterile saline per mg of mouse weight was used as control.

### Statistical Analysis

All data points denote biological replicates (i.e., no animal was used more than once for the same test), except for data comparing spontaneous and electrically stimulated seizures (Figure 1), which are technical replicates and the animal numbers are reported in the Figure 1 legend. All average data values are mean ± SEM. Statistics were computed using GraphPad Prism version 9 (GraphPad Software, Inc.) and comparisons were considered statistically detectable when *P* < 0.05. All data was evaluated for normality of residuals by the Shapiro-Wilk test prior to selecting a statistical test. Differences between two groups that passed normality testing were assessed by unpaired, 2-tailed Student’s *t*-test and differences between two groups that did not pass normality testing were assessed by Mann-Whitney test. Differences between more than two groups were assessed by 1- or 2-Way ANOVA followed by Tukey’s or Sidak’s multiple comparison tests, respectively. All data assessed by ANOVA passed normality testing. Comparison of survival proportions were done using a one-sided Fisher’s exact test. Effect size is reported as Cohen’s d^1^ for all significant comparisons of two groups.

## Results

### Comparison of Stimulated and Spontaneous Seizure Phenotypes

In order to administer autonomic agents proximal to seizures, we needed to temporally control seizure occurrence in our mice. To accomplish this, we used hippocampal electrical stimulation protocols similar to those used in kindling experiments to reliably, and repeatedly, induce seizures on command in D/+ mice. We compared cardiac and respiratory parameters between these electrically stimulated seizures and the spontaneous seizures experienced by D/+ mice (Figure 1). Both spontaneous and stimulated seizures had similar peri-ictal semiology and were qualified as tonic seizures; they were dominated by an extended tonic phase noted by hindlimb extension, large amounts of EMG activity, and apnea (Figures 1A,B). Pre-ictal heart rate was normocardic (i.e., between 500 and 600 bpm) for both seizure types (Figures 1A_1_,B_1_), and was followed by ictal bradycardia (Figures 1A_2_,B_2_) and postictal tachycardia (Figures 1A_3_,B_3_). Both spontaneous and stimulated seizures had apnea that was coincident with the tonic phase (Figures 1A_2_,B_2_). Overall, heart rate (HR) and respiratory frequency (Rf) were very similar (Figures 1C,D), with the exception that both parameters were elevated immediately prior to stimulated seizures for HC stimulated mice. We attribute this to the fact that HC stimulated mice were placed in the chamber shortly before stimulation and the experimenters were present and adjusting the chambers during the entirety of the experiment, likely causing an increased state of arousal in the mice. All recorded spontaneous seizures occurred when experimenters were absent. The relative timing of the ECoG and tonic phase initiation was different between the seizure types (Figure 1E). However, both the ictal and apnea durations of stimulated seizures were shorter than those of spontaneous seizures (Figures 1F,G). Thus, while spontaneous and HC stimulated seizures have some differences, the behavioral classification and cardiorespiratory parameters are essentially identical, making them suitable for the current study.

### Evaluation of Autonomic Pharmacology

Pharmacological agents used to manipulate β-adrenergic (i.e., 10 mg/kg sotalol and 50 mg/kg isoproterenol) and cholinergic muscarinic receptors (i.e., 1 mg/kg atropine and 0.5 mg/kg carbachol) were dissolved in sterile saline and injected intraperitoneal 10–15 min prior to successful seizure stimulation. The timing and dosages were chosen based on preliminary results (data not shown) and previous reports demonstrating successful blockade and stimulation of the receptors (Rose et al., 2007; Zhan et al., 2009; Benes et al., 2012; Kim et al., 2018; Wengert et al., 2021). Based on preliminary experiments where these agents were injected i.p. and heart rate was monitored (data not shown), it was determined that the optimal time to stimulate seizures was 10–15 min after injection. To assess drug effectiveness immediately prior to electrical seizure stimulation, we compared the geometric mean and standard deviations (SD1 and SD2) of the RR interval prior to seizure stimulation (Supplementary Figures 1A–C). Sinus arrhythmia is the main source of heart rate variability and is dependent on cardiac muscarinic receptors (Billman, 2011). As expected, when atropine (1 mg/kg i.p.) was administered, SD1 and SD2 decreased, in addition to a non-statistically significant RR decrease (Supplementary Figure 2A).

Conversely, the muscarinic agonist, carbachol (0.5 mg/kg i.p.), more than doubled the RR interval and increased SD1 and SD2 (Supplementary Figure 2B). Although it did not reach our statistical threshold of α = 0.05, as expected sotalol (10 mg/kg i.p.) increased and isoprenaline (50 mg/kg i.p.) decreased RR interval (Supplementary Figures 2C,D).

We administered high doses of the pharmacological agents to ensure that blockade and activation of the receptors was maximal. At these dosages, many of the agents may have central, in addition to peripheral affects. An advantage to our approach of electrical stimulation was the ability to determine ADTs generated under different drug conditions. None of the agents used had any noticeable effect on seizure threshold (Supplementary Figure 3A). In addition, seizure duration, as measured from first to last cortical spike wave, was not detectably affected (Supplementary Figure 3B). Taken together, the autonomic pharmacology had minimal effect on seizure semiology.

### Parasympathetic Drive to the Heart Is Maximized During the Tonic Phase

**FIGURE 2:**
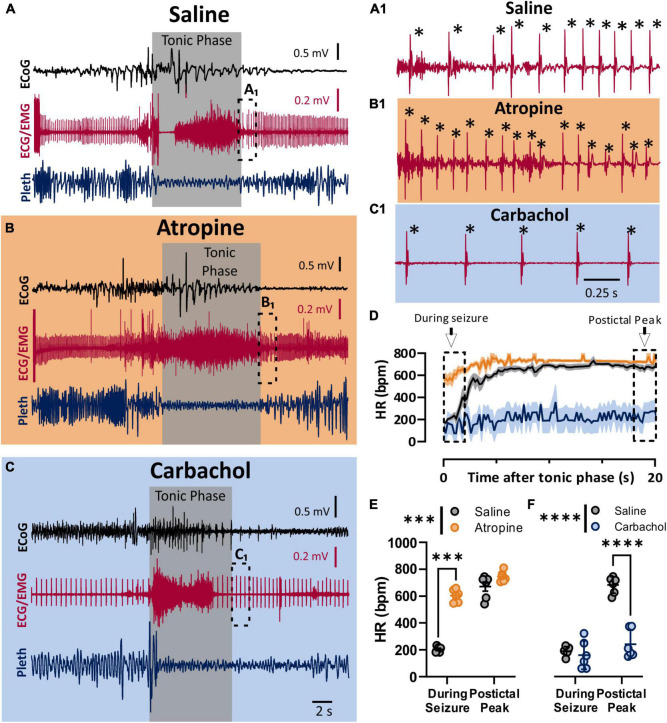
Parasympathetic activity is maximally activated during seizures. **(A–C)** ECoG, ECG, and breathing recordings during HC stimulated seizures 10–15 min after injection of saline **(A)**, atropine (1 mg/kg i.p.; **B**), or carbachol (0.5 mg/kg; **C**). **(A_1_,B_1_,C_1_)** Expanded ECG traces from end of tonic phase (dotted boxes in **A–C**). Note the frequency of R-waves is initially low for the saline, but not for the atropine condition. * Indicates identified R-waves. **(D)** Mean (lines) and SEM (shaded) of heart rate for 20 s after the tonic phase ended for saline (black), atropine (orange), or carbachol (blue) conditions. **(E)** Atropine increased heart rate during the seizure, but did not affect the postictal peak (*p* = 0.0006 and 0.2865, and *d* = 11.09 and 1.14, respectively; Sidak’s multiple comparison after significant 2-way ANOVA, *p* < 0.0001, *F*_(1,5)_ = 407.6, *n* = 6 mice). **(F)** Carbachol did not affect heart rate during the seizure, but did cause a detectable lower postictal peak (*p* = 0.5752 and < 0.0001, and *d* = 0.38 and 5.15, respectively; Sidak’s multiple comparison after significant 2-way ANOVA, *p* < 0.0001, *F*_(1, 5)_ = 208.8, *n* = 6 mice). ***p < 0.001 and ****p < 0.0001.

To assess the role of the parasympathetic nervous system in peri-ictal heart rate fluctuations, we administered either atropine (1 mg/kg i.p.), carbachol (0.5 mg/kg i.p), or saline control 10–15 min prior to seizure stimulation. The behavioral phases of the seizures were identical between the treatment groups, with initial wild running followed by a prominent tonic phase (Figures 2A,B). The tonic phase was coincident with apnea and a large amount of muscle activity apparent in the ECG recording. To estimate the heart rate during the seizure, we assessed the heart rate at the very end of the tonic phase, where R waves could be detected above the muscle activity (Figures 2A1,B1,C1). After injection with saline, heart rate at this time point showed clear ictal bradycardia (∼200 bpm), which developed into postictal tachycardia (∼650 bpm) within several seconds (Figure 2D, black line). Atropine treatment detectably increased ictal HR (Figure 2E). Conversely, carbachol had minimal effect on ictal HR but decreased postictal HR (Figure 2F).

### Sympathetic Drive to the Heart Is Maximized During the Postictal Phase

**FIGURE 3:**
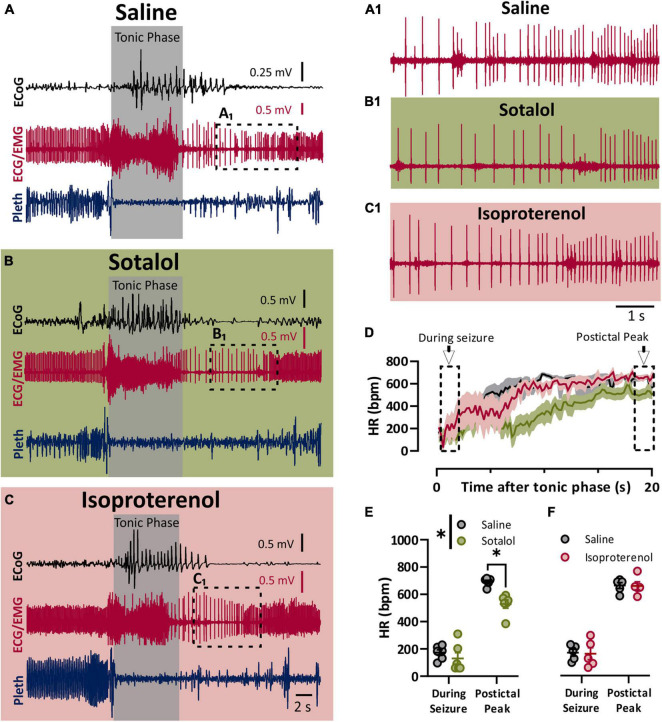
Sympathetic activity is maximally activated postictal. **(A–C)** ECoG, ECG, and breathing recordings during a HC stimulated seizure 10–15 min after injection of saline **(A)**, sotalol (10 mg/kg; **B**), or isoproterenol (50 mg/kg; **C**). **(A_1_,B_1_,C_1_)** Expanded ECG traces from end of tonic phase (dotted boxes in **A–C**). **(D)** Mean (lines) and SEM (shaded) of heart rate for 20 s after the tonic phase ended for saline (black), sotalol (green), or isoproterenol (purple) conditions. **(E)** Sotalol had no detectable effect on heart rate during the seizure, but did decrease the postictal peak (*p* = 0.6796 and 0.0471, and *d* = 0.53 and 2.89, respectively; Sidak’s multiple comparison after significant 2-way ANOVA, *p* = 0.0222, *F*_(1, 5)_ = 10.68, *n* = 6 mice). **(F)** Isoproterenol had no effect on heart rate during or after the seizure (*p* = 0.9811 and 0.6632, respectively; Sidak’s multiple comparison test after 2-way ANOVA, *p* = 0.8905, *F*_(1, 4)_ = 0.0215, *n* = 5 mice). *p < 0.05.

To assess the role of the sympathetic nervous system in peri-ictal heart rate fluctuations, we administered either sotalol (10 mg/kg i.p.), isoproterenol (50 mg/kg i.p), or saline control 10–15 min prior to seizure induction. Once again, the behavioral phases of the seizures were identical between the treatment groups, with initial wild running followed by a prominent tonic phase (Figures 3A–C). To estimate the heart rate during the seizure, we assessed the heart rate at the very end of the tonic phase, where R waves could be detected above the muscle activity (Figures 3A1,B1,C1). After injection with saline, heart rate at this time point showed clear ictal bradycardia (∼200 bpm), which developed into postictal tachycardia (∼650 bpm) within several seconds (Figure 3D, black line). Sotalol treatment had a negligible effect on ictal HR but reduced postictal HR (Figure 3E). Isoproterenol had minimal effect on either ictal or postictal HR (Figure 3F).

### Autonomic Pharmacology Has Minimal Effect on Breathing

**FIGURE 4:**
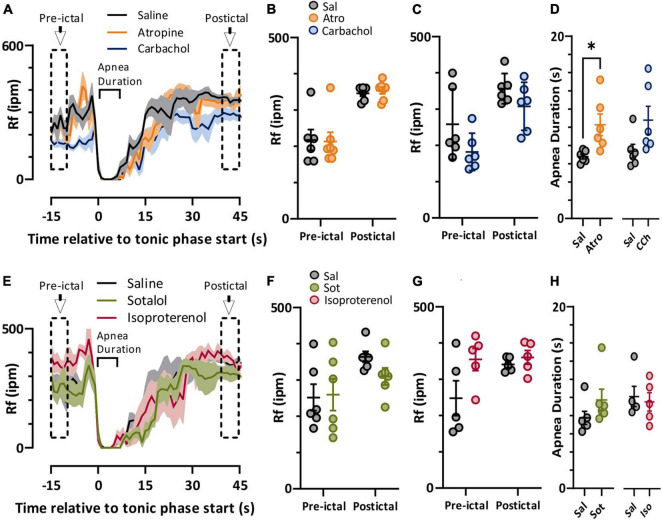
Autonomic agents had minimal impact on peri-ictal breathing. **(A)** Mean (lines) and SEM (shaded) of peri-ictal breathing rate when saline (black), atropine (orange, *n* = 6 mice), or carbachol (blue, *n* = 6 mice) was injected 15 min prior to seizure stimulation. **(B,C)** No difference in breathing rate was observed either prior to or after stimulated seizures for atropine (treatment factor of 2-way ANOVA, p = 0.9147, *F*_(1, 11)_ = 0.0120) or carbachol (treatment factor of 2-way ANOVA, *p* = 0.0924, *F*_(1, 5)_ = 4.315) treatments compared to saline. **(D)** Tonic phase apnea duration was increased by atropine (*p* = 0.0350, paired *t*-test, *t* = 2.869, df = 5, *d* = 1.66) but not carbachol (*p* = 0.0671, paired *t*-test, *t* = 2.331, df = 5, *d* = 1.15) treatments increased. **(E)** Mean (lines) and SEM (shaded) of peri-ictal breathing when saline (black), sotalol (green, *n* = 6 mice), or isoproterenol (purple, *n* = 5 mice) was injected 15 min prior to seizure stimulation. **(F,G)** No difference in breathing rate was observed either prior to or after stimulated seizures for sotalol (treatment factor of 2-way ANOVA, *p* = 0.5506, *F*_(1, 5)_ = 0.4091) or isoproterenol (treatment factor of 2-way ANOVA, *p* = 0.1626, *F*_(1, 4)_ = 2.920) treatments compared to saline. **(H)** Tonic phase apnea duration was not affected by either sotalol (*p* = 0.2302, paired *t* test, *t* = 1.366, df = 5) or isoproterenol (*p* = 0.7617, paired *t*-test, *t* = 0.3247, df = 4). *p < 0.05.

In both spontaneous and stimulated seizures breathing was compromised during the tonic phase (Figure 1). We compared pre-ictal and postictal respiratory frequency (Rf) and ictal apnea duration for the parasympathetic (Figure 4A) and sympathetic (Figure 4E) pharmacological agents. For the parasympathetic agents atropine and carbachol, there was no effect on pre-ictal or postictal Rf (Figures 4B,C). Interestingly, apnea was moderately longer for atropine-treated mice compared to control (Figure 4D). For the sympathetic agents sotalol and isoproterenol, neither pre-ictal Rf, postictal Rf, or apnea duration were noticeably different compared to saline control (Figures 4F–H).

### Postictal Bradycardia Is Not Sufficient to Produce Death

**FIGURE 5:**
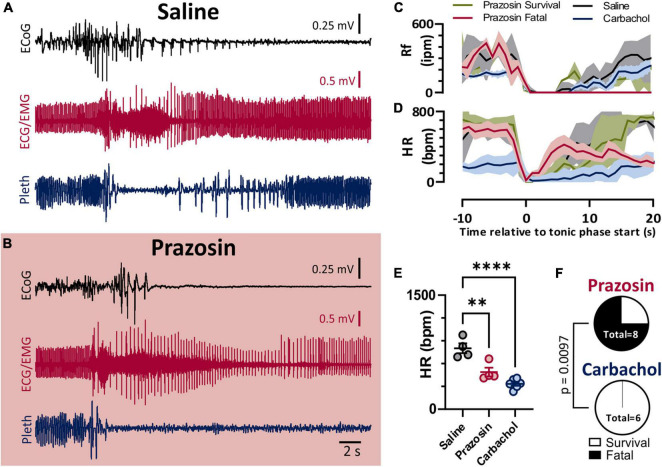
Blockade of alpha-1 adrenergic receptors drives non-fatal seizures into fatal seizures. **(A,B)** ECoG, ECG, and breathing recordings during an HC stimulated seizure after injection of saline **(A)** or prazosin (1 mg/kg; **B**). **(C)** Mean (lines) and SEM (shaded) of peri-ictal breathing rate when saline (black, *n* = 4), prazosin survival (green, *n* = 2), prazosin fatal (maroon, *n* = 4), or carbachol (blue, *n* = 5) was injected 15 min prior to seizure stimulation. **(D)** Mean (lines) and SEM (shaded) of peri-ictal heart rate when saline (black), prazosin (green for survival and purple for fatal), or carbachol (blue) was injected 15 min prior to seizure stimulation. **(E)** Peak postictal heart rate was lower for seizures induced after injection of prazosin or carbachol compared to saline (*p* = 0.0028 and *p* < 0.0001, and *d* = 2.53 and 4.57, respectively; Tukey’s multiple comparison test after 1-way ANOVA, *p* < 0.0001, *F*_(2, 11)_ = 25.96). **(F)** Proportion of fatal and non-fatal seizures under conditions of prazosin and carbachol injection (*p* = 0.0097, Fisher’s Exact Test). **p < 0.01 and ****p < 0.0001.

As we previously observed with audiogenic seizures in the D/+ mice (Wengert et al., 2021), administration of the α1 adrenergic receptor-antagonist prazosin (1 mg/kg i.p.) significantly increased the likelihood of seizure-induced death (Figure 5). Prazosin injection 10–15 min prior to seizure stimulation detectably increased fatality of seizure; 6 of 8 prazosin-injected mice died compared to zero out of 20 mice injected with saline with similar timing (*p* < 0.0001; Fisher’s Exact Test). Once again similar to our previous findings for both spontaneous audiogenic seizures in *Scn8a* mutant mouse models (Wengert et al., 2021; Wenker et al., 2021), breathing ceased during the tonic phase of electrically stimulated seizures in D/+ mice, and death occurred when breathing did not immediately recover postictally (Figure 5C). Postictal recovery of heart rate was muted for prazosin-injected fatal seizures (Figure 5D). Although postictal peak heart rate was less in both Prazosin and carbachol treated mice (Figure 5E), only prazosin treatment resulted in seizure fatality (Figure 5F).

## Discussion

We demonstrate that ictal bradycardia is due to maximal stimulation of the parasympathetic nervous system and postictal tachycardia is due to both reduction of the parasympathetic and stimulation of the sympathetic nervous systems. Furthermore, although we have previously shown that postictal tachycardia is associated with seizure survival (Wenker et al., 2021), preventing recovery of the postictal heart rate is not sufficient to cause fatality.

### Autonomic Mechanisms of Peri-Ictal Changes in Heart Rate

Ictal bradycardia is proposed to be due to activation of the parasympathetic nervous system, and is implicated in SUDEP (Benditt et al., 2015). Earlier work in anesthetized cats (Doba et al., 1975) and in kindled rats (Goodman et al., 1990, 1999), found that atropine could prevent ictal bradycardia. The studies in kindled rats observed bradycardia develop as seizures developed to Racine Stage 2. For the same seizures, the authors also reported ictal hypertension that was somewhat concurrent with bradycardia, which was attributed to simultaneous activation of both arms of the ANS (Doba et al., 1975; Goodman et al., 1990, 1999). However, the seizures described in these studies were never described as tonic, unlike seizures in the D/+ mice, and did not appear to produce apnea, although it was not measured. Still, it is possible that simultaneous sympathetic and parasympathetic activity occurs during the electrically stimulated seizures experienced by our D/+ mice. We did not record blood pressure in the present study, and the effect of parasympathetic activation of the SA node could override that of the sympathetic activation, which is a phenomenon known to occur when parasympathetic activity is high (Levy and Zieske, 1969; Yang and Levy, 1984; Kawada et al., 1999). However, it is also possible that seizure spread to the brainstem specifically affects the parasympathetic system; the preganglionic parasympathetic neurons that control the heart are located in the brain, whereas preganglionic sympathetic neurons are located in the spinal cord. Furthermore, postictal sympathetic stimulation could be reflexive, that is, apnea and overexertion cause hypoxia/hypercapnia that drive reflexes that increase the sympathetic system activity (Guyenet, 2006).

Although the previous studies in cats and rats mentioned above are informative, they were performed in models with unknown relevance to SUDEP. In this study, we utilize a mouse model of *SCN8A* epileptic encephalopathy. The particular mutation (i.e., N1768D) in the *Scn8a* gene was originally identified from an *SCN8A* patient that died from SUDEP (Wagnon et al., 2015). Expression of this mutation recapitulates clinical features, including spontaneous convulsive seizures and seizure-induced death (Larsen et al., 2015; Wagnon et al., 2016). In addition, patients with *SCN8A* epilepsy often have bradycardia and even temporary asystole during tonic seizures (Trivisano et al., 2019; Negishi et al., 2021); precisely what we have observed during the tonic seizures of D/+ mice (Wengert et al., 2021; Wenker et al., 2021). Bradycardia and asystole have been observed in seizures of other etiologies as well (Nei, 2009). Thus, the results obtained in the current study are likely relevant to seizures experienced by many epilepsy patients.

We, and others, have previously reported the occurrence of bradycardia during convulsive seizures in mouse models of SUDEP (Kalume et al., 2013; Aiba and Noebels, 2015; Aiba et al., 2016; Kim et al., 2018; Dhaibar et al., 2019; Loonen et al., 2019; Wengert et al., 2021; Wenker et al., 2021). In studies utilizing Dravet Syndrome mouse models, muscarinic antagonists prevented both bradycardia and death from thermally induced seizures (Kalume et al., 2013; Kim et al., 2018). In these studies, it is difficult to know whether the prevention of bradycardia was due to inhibition of parasympathetic input to the heart or simply that the mice survived. In fact, in Kim et al. (2018) when atropine was administered i.c.v. bradycardia and death were still prevented, suggesting that bradycardia is a feature of death, not necessarily parasympathetic activity.

### Autonomic Mechanisms of Seizure-Induced Death

Bradycardia has been proposed as a mechanism of seizure-induced death (Auerbach et al., 2013; Kalume et al., 2013). However, in our study, persistent bradycardia is not sufficient to produce death from a seizure. For example, suppression of heart rate with carbachol was not sufficient to produce death from electrically stimulated seizures. Furthermore, blocking of the sympathetic-induced tachycardia with sotalol did not prove fatal. This suggests that ictal cardiac function in isolation is not a major factor of whether a seizure will be classified as a fatal seizure or not.

As we have shown before with audiogenic seizures in D/+ mice (Wengert et al., 2021), administration of prazosin significantly reduces seizure survival. Prazosin is routinely used to inhibit sympathetic mediated increases in blood pressure (Stanaszek et al., 1983; Gross et al., 2005; Okuyama et al., 2018). A sympathetic-mediated increase in blood pressure could represent an important aspect of seizure survival. However, prazosin is also known to cross the blood brain barrier (Boehnlein and Kinzie, 2007; Taylor et al., 2008), and will also inhibit central α1 adrenergic receptors are important for arousal state (Perez, 2020), which have been implicated in seizure-induced death in other models (Kruse et al., 2019). Thus, based on our studies, it is unclear whether prazosin acts on central or peripheral adrenergic receptors to promote breathing recovery and survival.

### Effect of Atropine on Seizure-Induced Apnea

We, and others, have previously shown that seizure-induced apnea is a factor in fatality in mouse models of SUDEP (Faingold et al., 2017; Kim et al., 2018; Wengert et al., 2021; Wenker et al., 2021). Several lines of evidence suggest respiratory arrest is a driving factor of SUDEP. Apnea and oxygen desaturation have been reported in a large percentage of patients during and after convulsive and non-convulsive seizures (Nashef et al., 1996; Bateman et al., 2008; Lacuey et al., 2018a,b; Vilella et al., 2019a,b), and in 9 cases of SUDEP with adequate postictal cardiorespiratory monitoring, terminal apnea occurred prior to terminal asystole (Ryvlin et al., 2013).

Currently, mechanisms of fatal apnea are lacking; however, in a model of Dravet Syndrome it was found that seizure-induced apnea could be prevented by administration of atropine in a way that implicates central muscarinic receptors (Kim et al., 2018). In our hands with the *Scn8a* mutant mice, this was not the case. D/+ mice still experienced seizure-induced apnea when Atropine was administered. This could reflect different mechanisms of apnea in different models, or that seizures were invoked in different ways: heat-induced in Dravet Syndrome mice vs. hippocampal-stimulated in *Scn8a* mutant mice.

### Experimental Limitations

There are at least three limitations to our interpretations. First, we were only able to determine that persistent bradycardia was not sufficient to produce fatality from seizures. We could not validate whether it was necessary for fatal seizures, as we tested our hypotheses in a model where the induced seizures are routinely survived. Second, in the present study we only examined the ictal ECG of induced seizures. It remains possible that inter-ictal cardiac dysfunction plays a role in spontaneous seizure-induced death. Indeed, peri-ictal bradycardia was seen in two D/+ mice prior to spontaneous sudden death (Frasier et al., 2016). Third, ECG cannot determine the effectiveness of all cardiac function: for example, we do not know whether there is sufficient contraction and cardiac output. It is possible that these other forms of cardiac function play a role in mortality, and should be examined in future studies.

## Conclusion

Our findings suggest that extreme peri-ictal changes in heart rate are due to fluctuations in autonomic activity. However, these changes and are not, in themselves, a determining factor for seizure mortality in a model of genetic epilepsy. We also find that blockade of α1-adrenergic receptors is sufficient to produce fatality from seizures that are normally survived. Considering the sympathetic nervous system is activated postictal in survived seizures and could be acting on α1-adrenergic receptors of the vasculature, future directions understanding autonomic control of blood pressure could be pertinent.

## Data Availability Statement

The raw data supporting the conclusions of this article will be made available by the authors, without undue reservation.

## Ethics Statement

The animal study was reviewed and approved by the University of Virginia Animal Care and Use Committee.

## Author Contributions

IW and MP secured funding, designed experiments, and co-wrote the manuscript. EB and PW performed experiments and analyzed the results. All authors contributed to the article and approved the submitted version.

## Conflict of Interest

The authors declare that the research was conducted in the absence of any commercial or financial relationships that could be construed as a potential conflict of interest.

## Publisher’s Note

All claims expressed in this article are solely those of the authors and do not necessarily represent those of their affiliated organizations, or those of the publisher, the editors and the reviewers. Any product that may be evaluated in this article, or claim that may be made by its manufacturer, is not guaranteed or endorsed by the publisher.

## Footnotes

1
https://www.socscistatistics.com/effectsize/default3.aspx
