# Supplementary material for: Peri-Ictal Autonomic Control of Cardiac Function and Seizure-Induced Death
Source: Front Neurosci. 2022 Jan 21;15:795145. doi: 10.3389/fnins.2021.795145 (PMC8813980; doi:10.3389/fnins.2021.795145)
Supplement: Supplementary file 1 [file Data_Sheet_1.PDF]

Supplementary Material

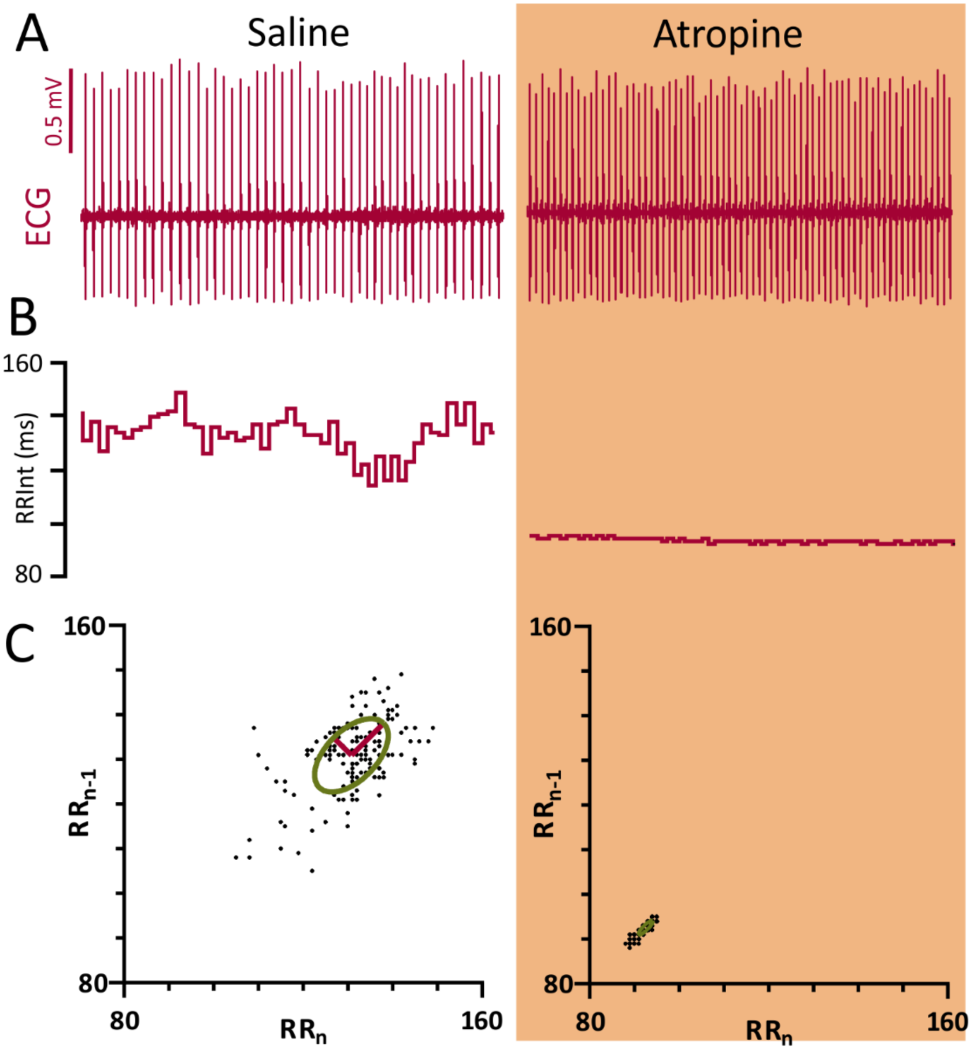

Supplementary Figure 1. Intraperitoneal atropine reduces heart rate variability in D/+ mice. A) Five seconds of pre-ictal ECG recording in a D/+ injected with either saline (left) or atropine (1 mg/kg i.p.; right) 15 minutes prior. **B)** RR intervals for ECG data in A. **C)** Poincare plots generated from 150 heart beats for saline and atropine conditions.

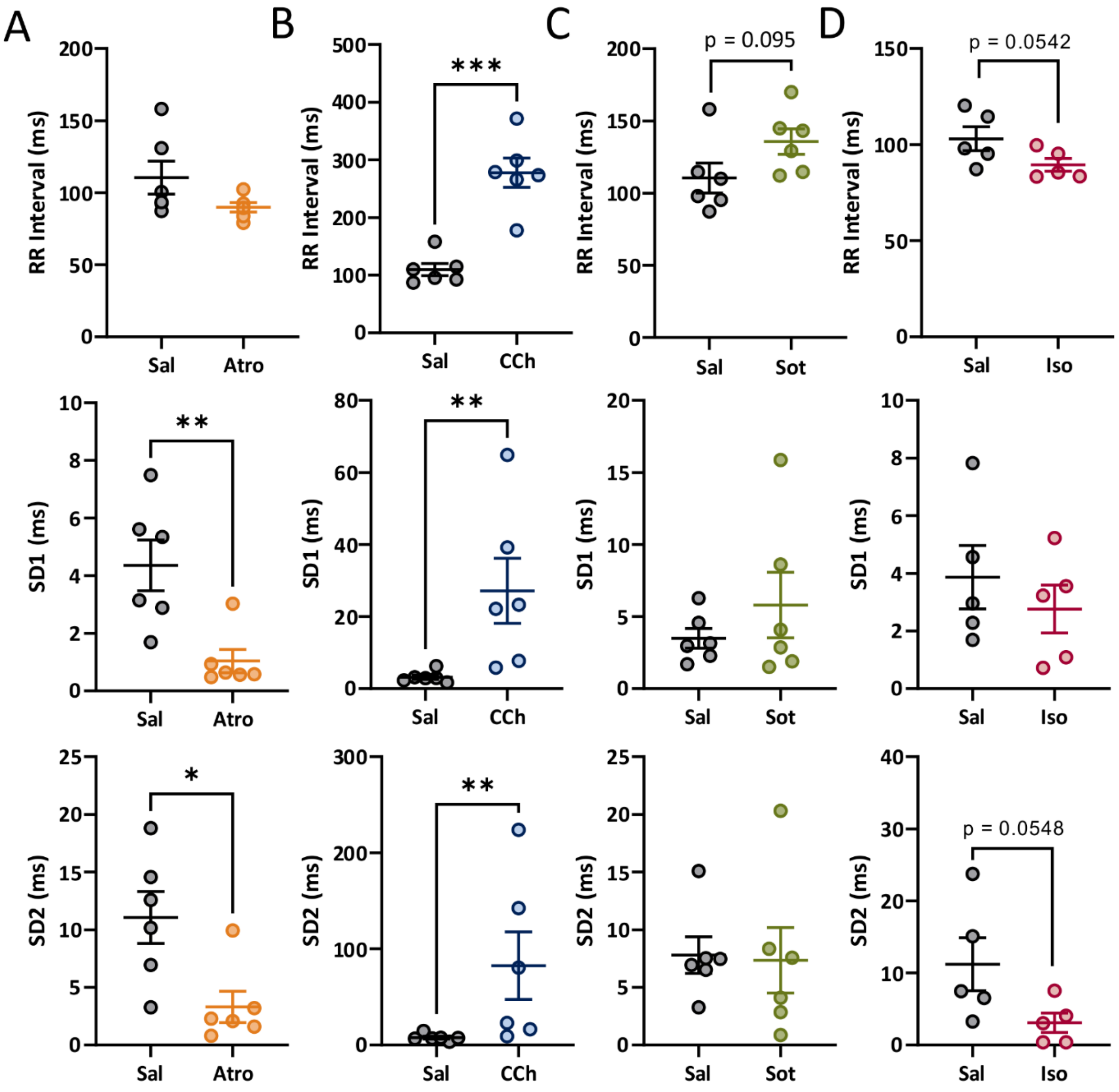

Supplementary Figure 2. Effects of other autonomic agents on pre-ictal heart rate variability. **A)** Mean RR interval was not detectably different between saline and atropine conditions (p = 0.1145, t = 1.729, df = 10, n = 6 mice). SD1 was detectably lower for atropine compared to saline (p = 0.0063, t = 3.443, df = 10, d = 1.99). SD2 was detectably lower for atropine compared to saline (p = 0.0149, t = 2.936, df = 10, d = 1.70). **B)** Carbachol (0.5 mg/kg i.p.) treatment detectably increased RR interval (p = 0.0001, t = 6.105, df = 10, d = 3.52, n = 6 mice) and SD1 (p = 0.0043, u = 1, d = 1.53), but not SD2 (p = 0.0043, u = 1, d = 1.23). **C)** Sotalol (10 mg/kg i.p.) treatment did not detectably effect RR interval (p = 0.0950, t = 1.844, df = 10, d = 1.06, n = 6 mice), SD1 (p = 0.9372, u = 17), or SD2 (p = 0.9372, u = 17). **D)** Isoproterenol (50 mg/kg i.p.) treatment did not detectably effect RR interval (p = 0.0542, t = 2.699, df = 4, d = 1.22, n = 5 mice), SD1 (p = 0.4477, t = 0.7984, df = 8), or SD2 (p = 0.0548, t = 2.688, df = 4, d = 1.31). * indicates $p < 0.05$.

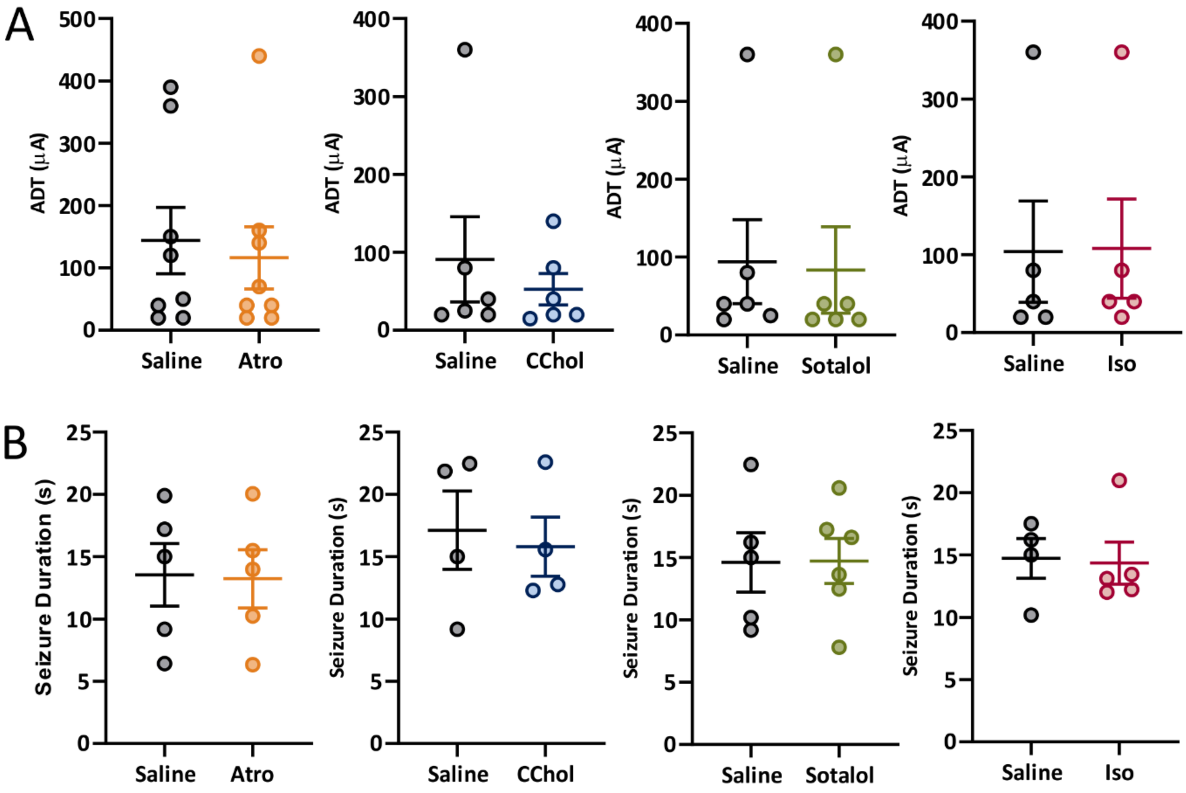

Supplementary Figure 3. Effects of autonomic agents on seizure duration and ADT level. A) Compared to saline control, ADTs were not different when mice were injected with 1 mg/kg atropine i.p. (Atro; $p = 0.8617$, $u = 30$, $n = 8$ mice), 0.5 mg/kg carbachol i.p. (CChol; $p = 0.6732$, $u = 15$, $n = 6$ mice), 10 mg/kg sotalol i.p. ($p = 0.4762$, $u = 13$, $n = 6$ mice), or 50 mg/kg isoproterenol i.p. (Iso; $p = 0.8810$, $u = 11$, $n = 5$ mice). All tests are paired t-tests. **B)** Compared to saline control, seizure duration was not different when mice were injected with atropine (Atro; $p = 0.9269$, $t = 0.0947$, $df = 8$), carbachol (CChol; $p = 0.7501$, $t = 0.3334$, $df = 6$), sotalol ($p = 0.9695$, $t = 0.0393$, $df = 9$), or isoproterenol (Iso; $p = 0.8776$, $t = 0.1598$, $df = 7$).
